# Characteristics, interventions, and outcomes of factitious disorder imposed on another (FDIA): a systematic review of 455 perpetrators and 469 victims

**DOI:** 10.3389/fpubh.2025.1723415

**Published:** 2026-01-20

**Authors:** Dan Wang, Mengzhen Zhao, Jiayi Yin, Yuanyuan La

**Affiliations:** 1 Center for Behavioral Health and School of Government, Beijing Normal University, Beijing, China; 2 Department of Social Medicine, Shanxi Medical University, Taiyuan, China

**Keywords:** factitious disorder imposed on another, hospital addiction syndrome, malingering by proxy, Munchausen syndrome by proxy, review

## Abstract

**Background:**

Factious disorder imposed on another (FDIA) is a severe yet often undetected form of abuse and mental disorder in which a caregiver recurrently falsifies a disease in another person to obtain medical and secondary benefits. We conducted this review to summarize the characteristics of both the perpetrators and the victims, as well as the interventions and outcomes.

**Methods:**

Seven electronic databases were searched for FDIA case reports and series published from the establishment of the database to August 20, 2024. Our search yielded 314 studies covering a total sample of 455 perpetrators and 469 victims. Information extracted included demographic and clinical characteristics of perpetrators and victims, in addition to interventions and outcomes.

**Results:**

Most of the victims were children (28.36%) and adolescents (27.93%), with slightly more males (56.50%) than females (43.50%). Most perpetrators were female (92.75%), married (52.09%), literate (30.33%), employed (26.59%), and with stable economic status (20.44%). Both the victims and perpetrators had physical, psychological, and behavioral problems. The most common intervention for FDIA was medical treatment, which was received by 25.49% of perpetrators and 40.51% of victims. The most common outcomes were victims being raised by foster care or social authorities (14.50%) and perpetrators being accused of a crime (29.67%).

**Conclusion:**

Our review opens the possibility of creating international multicenter databases of abuse cases in the FDIA context, which requires a multidisciplinary and transcultural approach to include all single case reports or case series to enrich the understanding of FDIA.

## Key Practitioner Message

FDIA perpetrators are predominantly female (93%), often with medical backgrounds (20%) and psychological disorders (e.g., somatization, depression).

Victims are primarily children (95%), experiencing physical/psychological harm; 15% enter foster care, while 8% die from abuse.

Early detection requires vigilance for symptom-perpetrator presence correlation (24% of cases).

Multidisciplinary interventions (medical, legal, psychological) are critical, with perpetrator prosecution rates reaching 30%.

International standardized databases are needed to improve FDIA identification and outcomes.

## 1 Introduction

Factious disorder imposed on another (FDIA)FDIA is a severe yet often undetected phenomenon in which a caregiver (typically a parent) recurrently falsifies a physical or mental illness to the persons under their care (typically a child) to seek medical support and psychological satisfaction (1). It is also called Munchausen by proxy syndrome (MBPS), factitious Disorder by Proxy (FDP), Munchausen’s syndrome by proxy (MSBP), or malingering by proxy (MBP) (2). The caregivers cause unnecessary and potentially harmful medical treatment to the persons under care through multiple ways, such as falsification of symptoms and tampering with specimens to meet their psychological needs (3). In this situation, the caregiver is the FDIA perpetrator, and the person to be cared for is the victim.

The name of MSBP comes from the 18th-century German officer Karl Friedrich von Munchhausen, who was notorious for creating false stories about himself and exaggerating his achievements (4). In ASHER (5), Dr. Richard Asher used Munchausen’s syndrome for the first time to describe a condition in which adults fabricated illnesses and gave fictitious medical histories about themselves in the pursuit of medical attention. In Meadow (6), MSBP was first described by Meadow in two families where the parents fabricated their children’s diseases by causing harm and then seeking medical treatment. Munchausen syndrome (MS) and MSBP are both factitious disorders; the former is fabricating diseases about oneself, while the latter is about other people.

Four concepts need to be clarified here. FDIA is a formal diagnostic term in DSM-5-TR (2022) and ICD-11 (6D51), defined as the perpetrator (usually a guardian) intentionally fabricating or inducing physical or psychological symptoms of others (such as children, older adults) in order to obtain psychological satisfaction (such as playing the role of a “needed caregiver”), with the motive being primary gain and no clear external reward. Munchausen Syndrome by Proxy is the historical name for this condition [proposed by Meadow (6)], which has now been abandoned by DSM-5-TR and ICD-11 and classified as FDIA. In contrast, Malingering by Proxy is not an official term in DSM or ICD, but rather an extension of “Malingering (V65.2)” based on DSM-5-TR, which refers to the perpetrator obtaining external benefits (such as medication, insurance compensation, and evasion of responsibility) by fabricating the symptoms of others, with the motive being secondary gain, and the perpetrator fully aware of the deceptive behavior (7). The core difference between the three lies in motivation (psychological satisfaction vs. material benefits) and terminology state (formal diagnosis vs. historical/derived concepts). Medical Child Abuse (MCA) is a clinical concept, which refers to medical abuse committed by guardians through forgery or direct causing of child diseases, emphasizing the harm to children (without limiting the perpetrator’s motive), and used in legal and pediatric protection scenarios (8). Due to the abandonment of the informal name “Munchausen Syndrome by Proxy” in DSM-5-TR, the FDIA has been uniformly used to emphasize the essence of behavior rather than historical labels. This study adopts the FDIA definition of DSM-5-TR and abandons old terminology (such as MSBP, MBP) to ensure consistency.

Surveys in recent decades have shown that the prevalence rates of FDIA have been increasing year by year, ranging from 0.002 to 0.53% (9–11). However, due to the disease’s concealment nature and difficulty in identification, FDIA is likely to be underdiagnosed and underreported (10, 12). FDIA perpetrators typically have maladaptive disorders or excessive attention-seeking in interpersonal relationships and present a wide range of illnesses. The clinical spectrum may include some common external manifestations, including cutaneous, neuropsychiatric, hematologic, infectious, and musculoskeletal signs or symptoms and external injuries. FDIA has been exceedingly described as rare, strange, and unimaginable cases involving various body parts and organ systems. In some serious instances, it may involve the induction of medical illness to the victims by administering poisonous substances or inaccurately applying medical measures to cause adverse consequences.

Being a form of abuse that presents a symptom complex, FDIA is also considered a severe form of abuse and child maltreatment involving a factitious disorder simulated and induced by a parent that can be life-threatening (39). Usually, parents or other caregivers bring the child to the hospital with complex symptoms that cannot be explained easily via physiological examination and general diagnosis, and these symptoms occur only when the child is with the caregivers (13). When the medical staff evaluates the child’s illness, they may harm the child by trying to treat the symptoms through physical or medical measures. This type of abuse exposes children to a substantial risk of severe harm due to numerous unnecessary medical examinations and treatments. Alarmingly, it is responsible for approximately 6 to 10 percent of fatalities among the children who fall victim to it (12, 14). The perpetrator may cause obvious suspected signs of illness on the child’s body through external force, lie about adverse reactions that the child may have before seeking medical treatment, or exaggerate and fabricate the child’s chief complaints. Additionally, the perpetrator may cause direct physical harm to the victims by overusing agents to induce symptoms. Consequently, surviving victims usually suffer from adverse health outcomes, such as longstanding psychological and neurocognitive disorders, which could yield triple harm, e.g., current, adult, and the next generation.

Although a plethora of case studies and reviews have been published on FDIA, the previous literature was predominantly focused on the characteristics of victims and perpetrators. So far, few reviews have summarized the characteristics of both the perpetrators and the victims and summarized the interventions and outcomes. Therefore, we conducted the current systematic review to present the characteristics of perpetrators, victims, and FDIA, as well as the available interventions on FDIA and their effects. Our findings would provide helpful clinical guidance in the prevention and management of FDIA. In addition, our study would direct future research on the prevalence, consequences, treatment strategies, and necessary legal reforms related to FDIA.

## 2 Method

This systematic review was conducted in accordance with Preferred Reporting Items for Systematic Reviews and Meta-Analysis (PRISMA) 2020 guidelines and did not require ethical approval as it analyzed only published data.

### 2.1 Data sources and search strategy

We searched FDIA cases or case series published from database establishment until August 20, 2024, from the following six English databases and one Chinese database: Web of Science, EBSCO-MEDLINE, PubMed, Science Direct, Scopus, Taylor & Francis, and the Chinese National Knowledge Infrastructure (CNKI). Although this review searched Chinese databases to reduce language bias, the final number of Chinese studies and cases included was very small. This indicates that FDIA’s research evidence is still highly concentrated in English literature, and more high-quality, cross-cultural studies are needed in the future to enrich global understanding. Our review results mainly reflect the FDIA features reported in English literature.

We used the following search terms: Munchausen syndrome by proxy or Munchausen by proxy or malingering by proxy or factitious disorder by proxy or factitious disorder imposed on another or fabricated illness or induced illness or hospital addiction syndrome or caregiver-fabricated illness or, pediatric condition falsification or medical abuse or malingering imposed on another or illness falsification or illness deception or factit or munchausen* AND case report or case series or case. The corresponding search formula for each database is shown in the Appendix Ι. We also searched Google Scholar, Qingli Research Service Literature Transfer Platform for additional studies. By contacting the corresponding authors, we obtained some of the original literature. However, due to the long publication period, knowledge payment, technical barriers, and other reasons, some full texts of the literature were still not found.

### 2.2 Eligibility criteria

We have developed detailed inclusion and exclusion criteria for literature screening. We included studies where FDIA was described using other terms, such as Factitious Disorder by Proxy, Munchausen syndrome by proxy, medical child abuse, child abuse in a medical setting, pediatric condition falsification, caregiver fabricated illness in a child, or Malingering by proxy. We included studies on FDIA that satisfied the following conditions: (1) Seek medical treatment in a clinical setting; (2) case report or case series; (3) report the demographic and/or clinical characteristics of the perpetrators and/or victims; (4) reported FDIA related treatment and outcomes. We adopted a conservative methodology and excluded cases where FDIA was only suspected. Cases were excluded if they did not describe the information of perpetrators and victims individually. Reviews and meta-analyses were excluded. We also excluded cases/studies that involved factitious disorder imposed on self.

### 2.3 Selection process

**Figure 1:**
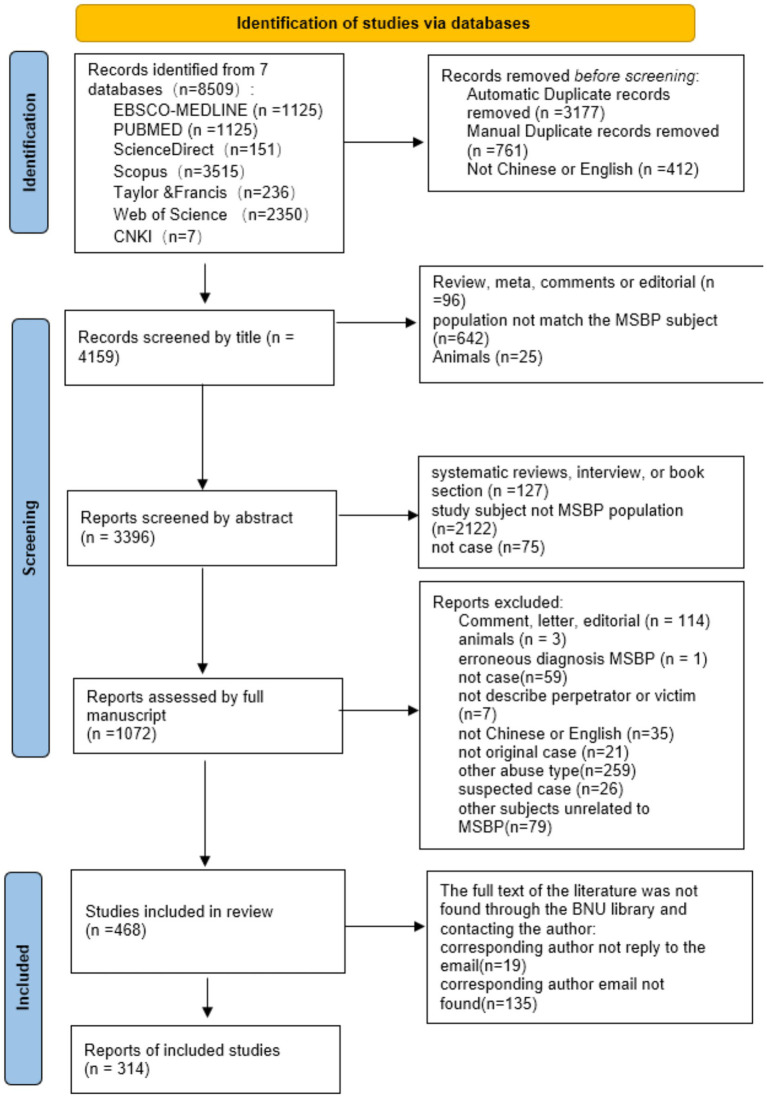
PRISMA flow diagram of included studies.

Studies were selected according to the Preferred Reporting Items for Systematic Reviews and Meta-Analyses (PRISMA) 2020 statement (15), and the flow chart of the search process is shown in Figure 1.

### 2.4 Data extraction

Two reviewers (DW and MZZ) independently extracted data from the included literature using Excel. Information extracted included the following five types: (1) Article information: article title, first author, publication year, and journal name; (2) case information: country, case number, the alternative name of FDIA, the relationship of perpetrator and victim, and the role of health professionals; (3) victim information: demographic characteristics, reasons for first medical treatment, method(s) of abuse, lasting time of abuse, diagnosed disease, and outcomes; (4) perpetrator information: demographic characteristics, clinical characteristics, perpetrator reported clinical characteristics of victims, and outcomes; (5) other information: the motivation for abuse, whether economic compensation had been received, and the role of video surveillance in identifying the cause. After two interviewers independently extracted data, a third reviewer (JYY) conducted cross-validation and resolved any discrepancy between the two individuals.

For multidimensional variables, only one most significant or relevant category is included in each case to avoid duplicate counting. The classification system includes a “Other” category to ensure that all observations are covered. This processing method aims to maintain the clarity and interpretability of the table, while highlighting the dominant distribution characteristics of each category. Treating “other” as an independent category also allows us to quantify those uncommon or atypical features, maintaining the integrity of the data presentation.

## 3 Results

### 3.1 Study characteristics

Information on 455 perpetrators and 469 victims was extracted from 314 studies. To partially mitigate selection bias, we included all eligible cases regardless of outcome severity. However, our results remain subject to the limitations of passive reporting systems. The date of publication for included studies ranged from 1977 to 2024. 147 (46.82%) studies came from North America (including Canada and the U.S.), 90 (28.66%) from Europe, 43(13.69%) from Asia, 16(5.10%) from South American, and the remaining came from Africa (*n* = 10, 3.18%) and Oceania (*n* = 8, 2.55%).

This study included a total of 314 studies. The distribution of case types was as follows: 301 (95.9%) were single perpetrator single victim cases, 12 (3.8%) were single perpetrator multi victim cases (mother and her children), and 1 (0.3%) was multi perpetrator single victim cases (parents and their son). In the data analysis, all individuals were independently included, so the sum of the total number of victims (*n* = 469) and perpetrators (*n* = 455) was greater than twice the total number of cases. This counting method aims to comprehensively reflect the number of individuals affected by violent incidents and is a common practice in such studies.

### 3.2 Demographic characteristics of victims and perpetrators

Table 1 shows the demographic information of the victims and perpetrators among cases with reported outcomes. The average age of the victims is 5.58 ± 9.10 years old, with a median of 3.0 years old. There were slightly more male victims (56.50%) than females (43.50%), and most of them were children and adolescents (95.31%). The average age of the perpetrators is 29.48 ± 9.57 years old, with a median of 28.0 years old. Most perpetrators were female (92.75%), married (52.09%), literate (30.33%), employed (26.59%), and with stable economic status (20.44%).

**Table 1:** Demographic characteristics of FDIA victims and perpetrators.

Variables	Categories	*N*/mean	%(±SD)
Victims			
Age		5.58	±9.10
Sex	Male	265	56.50%
	Female	204	43.50%
Population	Infant (<1)	80	17.06%
	Toddler (1–3)	103	21.96%
	Children (3–12)	133	28.36%
	Adolescent (12–18)	131	27.93%
	Adult (18–60)	14	2.99%
	The older adults (>60)	8	1.71%
Perpetrators			
Age		29.48	±9.57
Sex	Female	422	92.75%
	Male	33	7.25%
Marital status	Married	237	52.09%
	Unmarried	68	14.95%
	Divorced	26	5.71%
	Separated	18	3.96%
	Widowed	7	1.54%
	Not mentioned	99	21.76%
Educational level	Educated	138	30.33%
	Illiterate	58	12.75%
	Not mentioned	259	56.92%
Occupation	Employed	121	26.59%
	Unemployed	41	9.01%
	Not mentioned	293	64.40%
Economic status	Stable	93	20.44%
	Poor	38	8.35%
	Not mentioned	324	71.21%

### 3.3 Clinical characteristics of victims

Table 2 shows the clinical characteristics of the victims. The top five reasons for the victim’s first medical visit were abnormal symptoms and signs (19.40%), respiratory diseases (13.86%), digestive diseases (10.66%), blood and hematopoietic organ diseases (8.96%), and urinary and reproductive system diseases (6.61%). The victims suffered from psychopathology, with anxiety (17.06%), agoraphobia (11.51%), and somatization (6.40%) being the three most commonly reported mental illnesses. Moreover, they were involved in diverse behaviors that posed risks to their health, with the top five most common behaviors being physical abuse (13.22%), self-harm/suicide attempt (11.94%), and sexual abuse (6.82%), substance abuse (6.82%) and aggressive behavior (6.40%). These rates were calculated among published MBSP cases in included studies, though this may not reflect true population incidence.

**Table 2:** Clinic characteristics of FDIA victims.

Variables	Categories	*N*	%
Reasons for first medical treatment	Clinical and laboratory results of symptoms, signs, and abnormalities	91	19.40%
	Respiratory system diseases	65	13.86%
	Digestive system diseases	50	10.66%
	Blood and hematopoietic organ diseases	42	8.96%
	Urinary and reproductive system diseases	31	6.61%
	Neurological disorders	24	5.12%
	Circulatory system diseases	21	4.48%
	Injury, poisoning, and certain other results of external causes	14	2.99%
	Musculoskeletal and connective tissue Diseases	11	2.35%
	Pregnancy, childbirth, and postpartum period	10	2.13%
	Endocrine, nutritional, and metabolic diseases	10	2.13%
	Skin and subcutaneous tissue diseases	9	1.92%
	Infectious and parasitic diseases	9	1.92%
	Eye and accessory organ diseases	6	1.28%
	Ear and mastoid diseases	6	1.28%
	Congenital malformations, deformities, and chromosomal abnormalities	3	0.64%
	Tumor	3	0.64%
	Other	64	13.65%
Psychopathology	Anxiety	80	17.06%
	Agoraphobic	54	11.51%
	Somatization	30	6.40%
	Narcissistic personality disorder	27	5.76%
	Depression	25	5.33%
	Delusion	27	5.76%
	Other	6	1.28%
	Not mentioned	220	46.91%
History of hospitalization	≥3 times	340	72.49%
	<3 times	110	23.45%
	Not mentioned	19	4.05%
Health risk behavior	Physical abuse	62	13.22%
	Self-harm/suicide attempt	56	11.94%
	Substance abuse	32	6.82%
	Sexual abuse	32	6.82%
	Aggressive behavior	30	6.40%
	Smoking	20	4.26%
	Alcohol abuse	11	2.35%
	Not mentioned	226	48.19%

### 3.4 Clinical characteristics of perpetrators

Table 3 shows the clinical information of the perpetrators. Most perpetrators had a medical background (19.56%), and had experienced some traumatic events based on aggregated case reports, such as spousal extramarital affair (16.04%), divorce (8.35%), and physical abuse (5.49%). The perpetrators themselves also had physical, psychological, and behavioral problems. The top three somatic diseases were respiratory system diseases (12.75%), blood and hematopoietic organ diseases (5.93%), and infectious and parasitic diseases (5.27%). The top three psychopathologies were somatization (7.69%), depression (5.49%), and narcissistic personality disorder (2.64%). The top three health risk behaviors were self-harm/suicide attempt (16.48%), aggressive behavior (7.69%) and physical abuse (3.74%).

**Table 3:** Clinic characteristics of FDIA perpetrators.

Variables	Categories	*N*	%
Psychopathology	Somatization	35	7.69%
	Depression	25	5.49%
	Narcissistic personality disorder	12	2.64%
	Anxiety	10	2.20%
	Agoraphobia	9	1.98%
	Delusion	10	2.20%
	Other	5	1.10%
	Not mentioned	349	76.70%
Health risk behaviors	Self-harm/suicide attempt	75	16.48%
	Aggressive behavior	35	7.69%
	Physical abuse	17	3.74%
	Sexual abuse	14	3.08%
	Substance abuse	6	1.32%
	Alcohol abuse	3	0.66%
	Smoke	2	0.44%
	Other	12	2.64%
	Not mentioned	291	63.96%
Medical background	Yes	89	19.56%
	No	27	5.93%
	Not clear	339	74.51%
Somatic diseases	Respiratory system diseases	58	12.75%
	Blood and hematopoietic organ diseases	27	5.93%
	Infectious and parasitic diseases	24	5.27%
	Urinary and reproductive system diseases	19	4.18%
	Circulatory system diseases	18	3.96%
	Digestive system diseases	18	3.96%
	Neurological disorders	15	3.30%
	Musculoskeletal and connective tissue diseases	9	1.98%
	Endocrine, nutritional, and metabolic diseases	8	1.76%
	Skin and subcutaneous tissue diseases	5	1.10%
	Eye and accessory organ diseases	5	1.10%
	Ear and mastoid diseases	4	0.88%
	Tumor	2	0.44%
	Other	6	1.32%
	Not mentioned	237	52.09%
Traumatic event	Spousal extramarital affair	73	16.04%
	Divorce	38	8.35%
	Physical abuse	25	5.49%
	Sexual abuse	15	3.30%
	Sentence to prison	8	1.76%
	Miscarriage	3	0.66%
	Other	6	1.32%
	Not mentioned	287	63.08%

^**^All percentages are calculated as proportions of the total perpetrators sample (*N* = 455) unless otherwise noted.

### 3.5 Clinical characteristics of FDIA

Table 4 shows the clinical characteristics of FDIA in the analyzed case reports. Most FDIA occurred at home (50.32%), between mother and child (72.49%), and was motivated by emotional request (27.08%). Nearly fifth part of FDIA lasted for >12 months (18.55%). The top two reasons for suspected FDIA were “symptoms only appeared when the perpetrator was present” (23.88%) and “improvement when the perpetrator was absent” (23.03%). The top two methods of abuse were “fabricate or exaggerate symptoms” (13.22%) and poison (11.51%).

**Table 4:** Clinic characteristics of FDIA.

Variables		*N*	%
Relationship between perpetrators and victims	Mother and child	340	72.49%
	Father and child	16	3.41%
	Foster parent and child	13	2.77%
	Medical staff and patient	8	1.71%
	Husband and wife	5	1.07%
	Grandparents and child	3	0.64%
	Romantic relation	2	0.43%
	Other	82	17.48%
Reasons why FDIA suspected	Symptoms only appeared when the perpetrator was present	112	23.88%
	Improvement when the perpetrator was absent	108	23.03%
	Medical history clearly not matching the facts	58	12.37%
	Medical examination results inconsistent with symptoms	56	11.94%
	Witness of perpetration	25	5.33%
	Video surveillance discovered abnormal behaviors	10	2.13%
	Other	100	21.32%
Abuse setting	Home	236	50.32%
	Hospital	59	12.58%
	Other	174	37.10%
Lasting time	>12 months	87	18.55%
	7–12 months	24	5.12%
	3–6 months	35	7.46%
	<3 months	23	4.90%
	Not mentioned	300	63.97%
Methods of abuse	Fabricate or exaggerate symptoms	62	13.22%
	Poison	54	11.51%
	Physical injury	35	7.46%
	Multiple invasive diagnostic procedures	19	4.05%
	other	299	63.75%
Motivation	Emotional wish	127	27.08%
	Financial benefit	54	11.51%
	Other	288	61.41%

### 3.6 Intervention and outcomes

Table 5 summarizes the interventions for FDIA and the outcomes of perpetrators and victims. The most common intervention method was medical treatment and drug therapy, which was received by25.49% of perpetrators and 40.51% of victims. In addition, 20.04% of victims were separated from perpetrators. For victim outcomes, the largest proportion of victims were raised by foster care or social authorities (14.50%), followed by custody of other family members (10.87%) and death (7.89%). Regarding Perpetrator outcomes, nearly one-third (29.67%) were accused of a crime by the relevant legal authorities,25.93% were sentenced to imprisonment, and 24.40% were lost to follow-up due to the stigma associated with the condition, difficulties in accessing healthcare services, or the complexity of the cases.

**Table 5:** Intervention and outcomes of victims and perpetrators.

Variables	Categories	*N*	%
Victim outcomes	Foster care or social authorities	68	14.50%
	Custody of other family members	51	10.87%
	Death	37	7.89%
	Living with the perpetrator under the protection of authority	33	7.04%
	Lose to follow-up	12	2.56%
	Other	268	57.14%
Victim interventions	Victim receiving psychological assessment	22	4.69%
	Victims receiving medical treatment and drug therapy	190	40.51%
	Victims were separated from perpetrators	94	20.04%
	Other	163	34.75%
Perpetrator outcomes	Accused of a crime	135	29.67%
	Sentenced to imprisonment	118	25.93%
	Lost to follow-up	111	24.40%
	Suicide/self-injury	14	3.08%
	Other	77	16.92%
Perpetrator interventions	Perpetrator receiving psychological assessment	62	13.63%
	Perpetrator receiving medical treatment and drug therapy	116	25.49%
	Separation of victim and perpetrator	94	20.66%
	Other	183	40.22%

## 4 Discussion

### 4.1 Victims

Over a 47-year period of time (1977–2024), we found 314 studies of FDIA. FDIA victims in this review had similar characteristics to FDIA victims reported in previous research (16). Specifically, we found convergence in: victim demographics, with no pronounced sex bias [our study: 56.5% male; Fujiwara et al. (16): 47.6% male] and a concentration in early childhood (mean age: 4.6 VS 5.6 years); the core clinical presentation of recurrent, medically unexplained symptoms (e.g., suffocation, seizures); and a pattern of high medical utilization, with the majority of victims experiencing multiple hospitalizations prior to identification. Of the 469 victims whose sex was indicated, the sex of victims was roughly equal. Sheridan (14) found that 52% of victims were males and 48% were females among 415 FDIA children. Rosenberg (17) found that 46% of victims were males, 45% were females, and the remaining 9% were unidentified in 117 cases of FDIA. Therefore, the results were comparable across studies, indicating no significant sex differences. The victims were mainly young in our review, with 95.31% being children and adolescents. This result was similar to the proportion studied by others (14). The current data continues to indicate that FDIA victims are more frequently young people. It is noted that the victims often experienced psychological and behavioral issues.

### 4.2 Perpetrators

Our review showed that most of the perpetrators were females, the mothers of victims, literate, and had a medical background. Burton et al. (18) retrospectively identified 13 FDIA cases and concluded that most (62%) of the perpetrators were women, and many worked in healthcare. However, their medical background should be interpreted with caution since many perpetrators may lie about their occupation to gain the trust of the physicians. Psychological disorders were common in perpetrators, and our review showed that the top three most common psychiatric problems were somatization, depression, and personality disorders. This result was consistent with Yates and Bass’s (19) research. The difference was that this study mainly focused on narcissistic personality disorder, while their research primarily focused on borderline personality disorder. In addition, perpetrators also had some common co-occurring health risk behaviors, such as substance or alcohol abuse, aggressive behaviors, self-harm, and physical and sexual abuse, which was consistent with previous studies (20, 21). In our review, the majority of perpetrators reported spousal extramarital affairs and divorce. Another study showed that perpetrators reported the loss or separation of a parent at a young age (20). It is worth noting that the divorce of parents and the divorce of perpetrators is a double trauma.

### 4.3 Characteristics of FDIA

By far, the most common relationship between the perpetrator and victim of FDIA in the literature was the biological mother–child relationship. This review found that besides the mother–child relationship, the father-child relationship and the foster parent–child relationship are the next two largest groups. The relationship between the perpetrator and the victim is diverse and may also include grandparents, spouses, lovers, doctors, and patients, requiring careful identification. The literature lacks consistency in the therapeutic management and prognosis of FDIA. We found that disease fabrication most often resulted from falsifying or exaggerating symptoms that did not exist in victims and poisoning. Looking at the human-proxy cases in our review, the occurrence of abuse at home was more than four times higher than in hospitals. The initial place of abuse was at home, but the abuse continued until after hospitalization. The reason medical professionals suspected FDIA was that symptoms of victims only appeared when the perpetrator was present; once the perpetrator was absent, the victim’s condition would improve. Clinicians need to be cognizant of the relationship between the occurrence of symptoms and the presence of the perpetrator. Some medical facilities used covert video cameras in cases of suspected FDIA. This practice is controversial and poses many ethical questions. As shown in Table 4, the duration of FDIA exceeded 12 months in 87 cases (18.55% of those with data on this variable). Long-term abuse often causes severe physical and mental damage to the victims. FDIA is not primarily motivated by a desire for external rewards, such as economic gain, access to narcotics, or evasion of criminal responsibility. In our review, the motive for FDIA was mainly emotional requests, such as hoping to receive attention, longing for love, or out of some hatred. For those exhibiting typical medication acquisition behavior with unusual patterns, medications can also be a motivator, but this phenomenon was only present in the published literature for veterinary cases (22).

In addition, FDIA also involves some new variants. Vanelli (23) described an unusual case of web-mediated MBPS, which should be added to the already-known observation of Munchausen syndrome by the Internet in adults. A father who was addicted to health websites fabricated for his daughter a fictitious history of diabetes and gained access to medical protocols without any direct contact with a physician. Feldman (24) reported three similar cases in which people had access to Internet-based resources to fabricate or induce illnesses with the high credibility necessary to receive medical treatment. Another new variant is the comorbidity of Munchausen syndrome imposed on self and FDIA (25). Clin et al. (26) reported a case of a French mother who had presented with MS during her youth and transposed her self-aggressiveness onto her child, hence inflicting abuse. It is often difficult to accept the idea that a parent who feigns, exaggerates, aggravates, or self-induces physical and/or psychological illness or injury can deliberately induce suffering in his/her own child. Another similar case is a 27-year-old Caucasian woman who induced antepartum hemorrhage and rupture of membranes with a knitting needle at 26 weeks gestation, leading to delivery of the infant (27). The victim of self-induced preterm delivery survived, but he was subject to further abuse by his mother, suffering from MS.

### 4.4 Outcomes of victims and perpetrators

Once a diagnosis of FDIA is confirmed, a multidisciplinary approach is needed to achieve the best intervention effects. Some case reports focus on the use of pharmacological agents in the treatment of FDIA. Both analytical and cognitive-behavioral approaches have been used to treat FDIA (28). The victim’s safety is the most crucial consideration. Separation from the caregiver who is inducing illness can be diagnostic but must meet stringent criteria. Previous studies have shown that cases of MBPS had the best outcomes if the victims were taken into long-term care at a safe place without access to perpetrators (29). Once diagnosed with FDIA, the victim is most likely to be sent to a foster care center or taken care of by other guardians. If there are no other relatives or appropriate social institutions to take care of them, the victims will continue to live with the perpetrators under the protection and monitoring of relevant authorities, such as the Children’s Welfare and Protection Bureau, Social Assistance Center, and other institutions. After being diagnosed, most perpetrators were charged with committing crimes and went through intensive judicial procedures. Due to severe mental illness, the perpetrator may die by suicide. Existing research suggests that FDIA (Agency for Obsessive Compulsive Disorder) perpetrators generally exhibit severe psychopathological features, particularly borderline or performative personality disorders, depression, and trauma related disorders (19). These comorbidities significantly increase the risk of suicide for perpetrators, while a history of childhood trauma and pathological coping mechanisms (such as manipulative behavior or extreme emotion regulation strategies) may further exacerbate their tendency towards self-destruction (30). It is worth noting that when FDIA actions are exposed or face legal consequences, perpetrators may experience a sharp increase in suicide risk due to social isolation, shame, and psychological breakdown. Therefore, clinical intervention needs to focus on the suicidal tendencies of the perpetrator while assessing their mental state, especially during critical stages of case exposure or judicial proceedings, in order to develop targeted risk management strategies.

In managing fabricated or induced illness in children, the caregiver’s willingness to acknowledge concerns and accept some responsibility for harming the child, as underscored by Bass and Glaser (25), is pivotal for successful intervention. When caregivers embrace this responsibility, it fosters better communication with healthcare providers, leading to more accurate diagnoses and compliant treatment, which are essential for the child’s recovery. Conversely, resistance from caregivers can obstruct the management process, prolonging the child’s distress and increasing the risk of recurrence. Although our study did not directly focus on this aspect, it’s clear that caregiver acceptance could have influenced our case outcomes, indicating a need for further research and interventions to encourage such acknowledgment in future practices.

There are significant cross-border differences in the identification and handling of FDIA: (1) detection rate: High income countries (such as the United States and the United Kingdom) rely on child protection teams, while low-income areas may miss diagnosis (31); (2) legal consequences: Some countries (such as Sweden) tend to prioritize family restoration, while others (such as Australia) mandate criminal prosecution (32); (3) Cultural factors: In collectivist cultures, family reputation pressure may inhibit case reporting (33). Future studies should establish transcultural databases using standardized ICD-11 criteria to mitigate reporting biases.

The final number of cases included in this study (*n* = 455 perpetrators and 469 victims) differed from the number included in previous important systematic reviews, such as Yates and Bass (19) (*n* = 796 perpetrators). This difference is mainly due to the following carefully considered methodological choices: (1) Strict limitations on “clear diagnosis”: Unlike Yates and Bass (19) inclusion of “confirmed or highly suspected” cases, this study strictly limits the inclusion criteria to cases that have been confirmed as FDIA through medical or legal procedures to ensure homogeneity of the analyzed subjects and robustness of the conclusions. Therefore, we excluded case reports that only remained at “clinical suspicion” or “high likelihood” but lacked conclusive evidence [such as some cases in Bools et al.'s (34) study]. Although this choice reduces the sample size, it improves the diagnostic certainty of the included cases. (2) Requirement for ‘individual level data’: Our research aims to quantitatively synthesize demographic and clinical characteristics of both perpetrators and victims. Therefore, we require that the included studies provide traceable individual case data. Many large-scale case series studies (such as Bass et al. (20)) provide overall data but do not provide detailed descriptions of the characteristics of each case, which does not meet our data extraction requirements. The review by Yates and Bass (19) may include these studies to calculate the overall incidence rate, but its depth of analysis may be limited. (3) Requirement for “Treatment and Outcome” Reporting: One of the core objectives of this review is to evaluate FDIA interventions and outcomes. Therefore, our inclusion criteria (4) explicitly require studies to report relevant treatment or follow-up information. Some studies that only describe the diagnostic process or acute phase manifestations without involving any treatment or long-term outcomes were therefore excluded. We believe that this focus is necessary and reasonable for achieving our specific research objectives. In short, the difference in sample size is not an oversight, but rather stems from different research purposes and stricter inclusion criteria. Our approach aims to provide a more precise and in-depth analytical framework for the characteristics, treatment, and outcomes of FDIA cases with clear diagnosis. Of course, this inevitably comes with the cost of limited sample representativeness, which we have fully elaborated as a limitation of this study in the article.

### 4.5 Discussion and integrated implications

This study finds FDIA victims are mostly young children, with perceivers primarily female family members—underscoring the issue’s complexity that defies single-disciplinary or cultural solutions. We propose an integrated framework: (1) Multidisciplinary collaboration: Form a pediatrician-psychiatrist-led rapid response team (including social workers, child protection experts, medical legal advisors, and hospital safety officials). Pediatricians spot suspicious, non-medically justified symptoms; psychiatrists evaluate perceivers’ potential psychological disorders (e.g., affective, borderline personality disorders); social workers/child protection experts assess family ecology and create safety/long-term care plans; medical legal advisors ensure intervention compliance. This breaks information silos for seamless identification, diagnosis, and intervention. (2) Cross-cultural adaptation: While perceivers’ identities share transnational commonalities, their motivations, family dynamics, and help-seeking behaviors are culturally shaped (e.g., family honor-focused or mental illness-stigmatizing cultures may impede disclosure). Adapt interventions by: (a) Training staff to recognize cross-cultural atypical distress/somatic signs; (b) Partnering with community leaders/cultural intermediaries on destigmatized public education; (c) Ensuring reporting mechanisms/support services respect family diversity and offer culturally competent care.

The younger age of victims in this study and the family closeness of perceivers pose unique public health challenges. Young children are unable to self report and their health relies entirely on honest statements from their guardians, making the healthcare system the primary and sometimes the only protective barrier. Therefore, public health strategies must shift from “passive diagnosis” to “active protection.” Systematically elaborate on public health strategies: (1) Primary prevention: Educate prospective parents/caregivers on safe care and stress management to reduce parenting anxiety/knowledge gaps; frame help-seeking as strength, with non-judgmental maternal psychological support. (2) Secondary prevention: Conduct pediatric structured risk assessments for repeated visits/inconsistent symptoms, set EHR-based warnings for cross-institutional/specialty frequent medical visits, and establish Family Support Centers offering respite care, parenting training, and counseling for high-stress/pre-crisis families—prioritizing family support to end abuse. (3) Tertiary prevention: Provide victims with trauma-informed care, long-term follow-up, and legal support; deliver targeted therapy (CBT/DBT) to perpetrators with victim safety as the priority; and offer psychological assessments and counseling to siblings and other family members.

In summary, specific recommendations include: firstly, medical institutions should mandate FDIA training and develop multi departmental clinical pathways, clarifying roles and information sharing processes. Secondly, cultural competence should be incorporated into professional education and multilingual, culturally adapted family assessment tools should be developed. Finally, a mandatory cross departmental information disclosure and case conference system should be established, and longitudinal research aimed at identifying the most effective support models should be funded, with a focus on successful intervention cases in different cultural backgrounds.

### 4.6 Strengths and limitations

Although FDIA has been extensively studied in the literature, previous studies are predominantly focused on the victims, and much less is known about the perpetrators. Our review represents the first attempt to characterize both the perpetrators and victims comprehensively. In addition, we summarize the available evidence on the interventions for FDIA and the outcomes for both the perpetrators and victims. We systematically reviewed 314 articles and extracted data on 455 perpetrators and 469 victims of FDIA. Our results are in accordance with prior results in the literature, further substantiating the need to identify various patterns in the presentation of this form of abuse (38).

Nevertheless, our study has several limitations. First, although we investigated sociodemographic and clinical factors associated with FDIA, we did not examine the motivation underlying the abuse, the role of medical staff, and the treatment of the disease. Second, the literature search was limited to cases published in English and Chinese and may not represent findings in non-Western or non-Chinese backgrounds.

In response to the review comments and to enhance transparency, we hereby clarify that through the CNKI database (All authors are native Chinese speakers), we have retrieved a total of 2 Chinese studies that meet all inclusion criteria. These two studies are all published case reports, contributing individual data from two perpetrators and two victims. Therefore, although we included CNKI to reduce publication bias and expand geographical coverage, the incremental contribution of Chinese research to the final analysis sample of this review was approximately 0.44% (2/455) of the total number of perpetrators and 4.65% (2/43) of cases in Asia. This result indicates that although our retrieval strategy is inclusive, the main evidence base of this study still mainly comes from English literature databases, and the addition of CNKI did not substantially change the regional or linguistic composition of the samples. Third, publication bias could complicate this study, much like the “file-drawer problem” that the meta-analysis faced (35). Publication bias, likely amplified for the dramatic subject matter of FDIA, may have skewed the literature towards more sensational cases, affecting the generalizability of our findings. As such, conclusions should be interpreted with caution, and future research should focus on more objective data collection methods and minimizing publication bias to better understand this complex disorder. Finally, it is widely acknowledged that FDIA is significantly underdiagnosed and therefore underreported (30). Even though, due to our minimal resources, the full text of many case reports cannot be found.

Our findings must be interpreted in light of the inherent biases of case report literature. First, publication bias likely over represents rare or severe cases of MBSP, skewing prevalence estimates. Second, diagnostic variability across studies may inflate heterogeneity. Third, the absence of denominator data in case reports precludes calculation of true population-based rates. Thus, our statistics reflect frequencies within the published literature, not the actual epidemiology of MBSP. These limitations align with broader critiques of case report syntheses (36, 37).

## 5 Conclusion

FDIA is difficult to diagnose due to its varied clinical manifestations. Any illness could be the subject of falsification, including psychiatric disorders. A cycle of abuse and factitious illness develops when FDIA remains unidentified. Early recognition is the key to decreasing the morbidity and mortality associated with FDIA. Identifying and educating potential perpetrators may reduce the incidence of FDIA. Future studies should prioritize prospective registries with standardized diagnostic criteria to generate unbiased epidemiological estimates. Until such data exist, our synthesis provides a preliminary framework for understanding FDIA manifestations. Our review opens the possibility of creating international multicenter databases of abuse cases in the FDIA context. Based on these databases, it would be interesting to pool data on the characteristics of perpetrators and victims to understand whether a certain profile of FDIA uses a specific type of falsification to induce the illness. Last but not least, medical staff from different departments should be encouraged to contribute to the international databases. A multidisciplinary and transcultural approach is needed to include all single case reports or case series to enrich the understanding of FDIA.

## Data Availability

The original contributions presented in the study are included in the article/supplementary material, further inquiries can be directed to the corresponding author.

## Appendix

Appendix I Search strategy.

**Table:**

No.	Database	Search terms
1	EBSCO-MEDLINE	AB(“munchausen syndrome by proxy” or “munchausen by proxy” or “malingering by proxy” or “factitious disorder by proxy” or “factitious disorder imposed on another” or “fabricated illness” or “induced illness” or “hospital addiction syndrome” or “caregiver-fabricated illness” or “pediatric condition falsification” or “medical abuse” or “malingering imposed on another” or “illness falsification” or “illness deception” or factit* or munchausen*) AND AB(“case report” or “case series” or case)
2	PUBMED	(“munchausen syndrome by proxy”[Title/Abstract] OR “munchausen by proxy”[Title/Abstract] OR “malingering by proxy”[Title/Abstract] OR “factitious disorder by proxy”[Title/Abstract] OR “factitious disorder imposed on another”[Title/Abstract] OR “fabricated illness”[Title/Abstract] OR “induced illness”[Title/Abstract] OR “hospital addiction syndrome”[Title/Abstract] OR “caregiver-fabricated illness”[Title/Abstract] OR “pediatric condition falsification”[Title/Abstract] OR “medical abuse”[Title/Abstract] OR “malingering imposed on another”[Title/Abstract] OR “illness falsification”[Title/Abstract] OR “illness deception”[Title/Abstract] OR factit*[Title/Abstract] OR munchausen*[Title/Abstract]) AND (“case report”[Title/Abstract] OR “case series”[Title/Abstract] OR case[Title/Abstract])
3	Taylor & Francis	[[Abstract: “munchausen syndrome by proxy”] OR [Abstract: “munchausen by proxy”] OR [Abstract: “malingering by proxy”] OR [Abstract: “factitious disorder by proxy”] OR [Abstract: “factitious disorder imposed on another”] OR [Abstract: “fabricated illness”] OR [Abstract: “induced illness”] OR [Abstract: “hospital addiction syndrome”] OR [Abstract: “caregiver-fabricated illness”] OR [Abstract: “pediatric condition falsification”] OR [Abstract: “medical abuse”] OR [Abstract: “malingering imposed on another”] OR [Abstract: “illness falsification”] OR [Abstract: “illness deception”] OR [Abstract: factit*] OR [Abstract: munchausen*]] AND [[Abstract: “case report”] OR [Abstract: “case series”] OR [Abstract: case]]
4	Elsevier Science Direct	#1“Munchausen syndrome by proxy” or “Munchausen by proxy” or “malingering by proxy” or “factitious disorder by proxy” or “factitious disorder imposed on another” or “fabricated illness” or “induced illness” or “hospital addiction syndrome” or “caregiver-fabricated illness” or “pediatric condition falsification” or “medical abuse” or “malingering imposed on another” or “illness falsification” or “illness deception” #2 “case report” or “case series” or case
5	SCOPUS	(TITLE-ABS-KEY (“munchausen syndrome by proxy” OR “munchausen by proxy” OR “malingering by proxy” OR “factitious disorder by proxy” OR “factitious disorder imposed on another” OR “fabricated illness” OR “induced illness” OR “hospital addiction syndrome” OR “caregiver-fabricated illness” OR “pediatric condition falsification” OR “medical abuse” OR “malingering imposed on another” OR “illness falsification” OR “illness deception” OR factit* OR munchausen*) AND TITLE-ABS-KEY (“case report” OR “case series” OR case))
6	Web of science	“munchausen syndrome by proxy” OR “munchausen by proxy” OR “malingering by proxy” OR “factitious disorder by proxy” OR “factitious disorder imposed on another” OR “fabricated illness” OR “induced illness” OR “hospital addiction syndrome” OR “caregiver-fabricated illness” OR “pediatric condition falsification” OR “medical abuse” OR “malingering imposed on another” OR “illness falsification” OR “illness deception” OR factit* OR munchausen* (subject) and “case report” OR “case series” OR case (subject)
7	CNKI (China national knowledge internet)	(代理性孟乔森综合征or代理性佯病症 or 明肖森综合征 or 曼丘森综合症 or 孟乔森综合征 or 闽希豪生综合症) and(“病例报告” or “病例系列研究” or “病例” or “个案”)
